# Health-related quality of life of children and adolescents living with HIV in Lagos, Nigeria: a cross-sectional study

**DOI:** 10.11604/pamj.2022.41.344.23664

**Published:** 2022-04-28

**Authors:** Abideen Olurotimi Salako, Agatha Nkiruka David, Babasola Ayoola Opaneye, Kazeem Adewale Osuolale, Oluwatosin Olaseni Odubela, Priscilla Ngozi Ezemelue, Titilola Abike Gbaja-Biamila

**Affiliations:** 1 Clinical Sciences Department, Nigerian Institute of Medical Research, Lagos, Nigeria

**Keywords:** Quality of life, adolescent, anti-retroviral therapy

## Abstract

**Introduction:**

adolescents living with HIV [ALHIV] face the dual challenges of adolescence and coping with HIV infection. This study aims to evaluate health-related quality of life [HRQoL] of children and adolescents aged 8 - 18 years living with HIV in an HIV treatment centre in Lagos, Nigeria.

**Methods:**

we conducted a cross-sectional study among children and adolescents living with HIV and receiving antiretroviral therapy. HRQoL was assessed using the Paediatric Quality of Life Inventory [PedQoL™]. Socio-demographic data and HIV related clinical and laboratory characteristics were also obtained and tested based on HRQoL scores in order to determine if there were possible associations.

**Results:**

the study included 113 participants with a mean age of 14 (± 2.9) years. There was male predominance, with a male: female sex ratio of 1.1: 1. The mean duration of ART was 102.9 (±36.9) months and CD4 lymphocyte count was and 741.2 (±335.7) cell/mm^3^. The majority of participants (62%) were also virally suppressed. Based on self-reported data, the mean physical, psychosocial and total HRQoL scores were 85.0 [± 22.4], 78.5 [±17.5] and 81.6 [±18.4] respectively. Adolescents aged 13-18 years had significantly higher scores than children aged 8-12 years. Male patients who had been on ART for ≥60 months were also significantly associated with higher HRQoL scores (OR=5.46 [CI= 2.24-13.29], p = 0.0009) and OR= 4.80, [CI= 1.58 - 14.56] p = 0.0032).

**Conclusion:**

the majority of participants in the study had good HRQoL scores, attesting to the success of highly active antiretroviral therapy for HIV infection and the ease of access and availability to a comprehensive care.

## Introduction

The introduction of highly active antiretroviral therapy (HAART) has revolutionized the management of Human Immunodeficiency Virus (HIV) infection, turning a once fatal disease to a chronic, manageable condition [1]. Thus, children prenatally infected with HIV are now not only surviving into adolescence and adulthood but face reasonable prospects of a long and productive life. The survival and prospects however come at some cost, which include lifelong drug use, possible long-term side effects of the antiretroviral drugs, and the stigma and discrimination associated with being HIV infected [2]. Adolescents horizontally infected with HIV also face the same challenges.

Adolescence, (ages 10 years to 19 years), is the bridge period in human development between childhood and adulthood. It is characterized by hormonal changes, morphological transformations and psycho-social changes. It is a period of increasing independence, development of social skills and learnt behaviour modulated by peer groups and family, societal and religious norms. These could be quite challenging and have significant life-long impact [3, 4]. Adolescents and young people (ages 10 years to 24 years) bear an inordinate burden of HIV. In 2008, the United Nations Children´s Fund (UNICEF) reported approximately 200,000 new HIV infections in adolescents and an estimated 33,000 HIV related mortalities, with the majority of these occurring in Sub-Saharan Africa. This prompted UNICEF and UNAIDS, in partnership with other international agencies, to launch the “ALL IN! to END Adolescent AIDS Initiative” towards ending the AIDS epidemic among adolescents by 2030 [5, 6].

Although the burden of HIV in Nigeria has recently been revised downwards with the national prevalence at 1.4%, the burden of new infections and mortality remains high among adolescents and young adults [7, 8]. Adolescents living with HIV still face a myriad of health challenges (physical, social and mental) which impact on their quality of life [9, 10]. There have been varying reports on the impact of HIV/AIDS on the quality of life among children and adolescents living with HIV. While some studies reported a negative impact of the disease on life, other researchers have shown positive effects of HIV/AIDS on adolescent life. The differences in the reports could be due to a variety of reasons, which include the availability and accessibility of adolescent-friendly health care services, the stage of HIV/AIDS among affected adolescents, long-term disorders associated with HIV/AIDS and antiretroviral therapy, and the well-recognized challenges of adolescence [1, 2, 11-18].

The Federal Government of Nigeria rolled out the HIV/AIDS Treatment programme in 2002 in 25 health facilities across the country. Since then, there has been widespread scale-up of the programme. In the almost two decades of this programme in the country, there have been few reports on the health-related quality of life (HRQoL)of people infected with HIV [19-22], and most of the studies have focused on adults. There is thus a paucity of information on the HRQoL of children and adolescents living with HIV in Nigeria. This study therefore set out to evaluate the health related quality of life (HRQoL) of children and adolescents (8-18years) living with HIV in Lagos, Nigeria, using the Paediatric quality of life questionnaire (PedsQL™).

## Methods

**Study design/site:** this was a cross-sectional study carried out over a four-month period (June to September 2019) at the HIV Clinic of the Clinical Sciences Department at the Nigerian Institute of Medical Research (NIMR), Lagos, Nigeria. The Clinic offers comprehensive HIV care and treatment services to adults, adolescents, children and pregnant women and has a cumulative enrolment since inception in 2002 of over 25,000 patients. There is a cumulative paediatric and adolescent enrolment of about 1,600 since commencement of paediatric HIV services in 2004. Paediatric clinics take place once every week, while the Adolescent Clinic is on the second Saturday of every month. The clinic also has an adolescent club, which is open to all adolescents who know their HIV status. The club, coordinated by healthcare personnel in the Paediatric Unit (doctors, nurses, counsellors), meets immediately after the adolescent clinic. Saturday was intentionally chosen to avoid interference with the adolescent´s school activities and ensure their maximal attendance. The club provides a platform for interaction and discussion among the adolescents and coordinators, as well as occasional facilitators brought in to discuss issues of interest to the members, such as educational/career goals, sexual reproductive health issues, marital and fertility aspirations, ARV adherence challenges, and issues around disclosure, stigma and discrimination, among other things. All services at the paediatric and adolescent clinics are also provided free of charge.

**Study population:** one hundred and thirteen (113) children and adolescents aged 8-18 years living with HIV/AIDS, who have been on ART for at least six months and who gave informed consent/assent participated in the study. Those with co-morbid illnesses, such as Seizure disorders, Sickle cell anaemia and Hepatitis B/C, were excluded from the study.

**Study procedure:** children and adolescents attending the clinic were randomly selected from the clinic database (using a table of random numbers generated by the biostatistician) and assessed for eligibility for the study. The study was described to those who met the study criteria and their caregivers by a member of the study team [clinician and designated counsellor]. Those who agreed to participate were taken through a detailed informed consent and assent process culminating in their signing the informed consent/assent form, and were thereafter enrolled into the study. The case record form (CRF) designed for the study was used to collect data on sociodemographic characteristics and HIV related characteristics. The quality of life assessment for the study participants was done using a pre-tested interviewer-administered questionnaire; the Paediatric quality of life questionnaire (PedsQL™) [23], already validated among children of various cultures and countries including Nigeria [24-26]. The PedsQL™ questionnaire is a robust validated 23 generic core scale designed to measure the core dimensions of health, which are physical functioning (8), emotional functioning (5), social functioning (5) and school functioning (5). For children 8-12 years´, parental report forms were filled by the parents/caregivers. Before study commenced, permission to use the questionnaire was obtained from copyright owners [20]. The following definition of terms were used; “Good Quality of Life = Overall Physical/Psychosocial HRQoL aggregate score of 80%-100%, “Intermediate Quality of Life = Overall Physical/Psychosocial HRQoL aggregate of 60% - <80%, and “Poor Quality of Life = Overall Physical/Psychosocial HRQoL aggregate score of <60%.

**Data analysis:** collected data were entered into an Excel spreadsheet, cleaned and transferred onto an SPSS spreadsheet for analysis with SPSS version 23.0. Categorical data were summarized using proportions, while continuous data were checked for normality assumption. Normally distributed data were presented as mean with standard deviation, while the skewed data were summarized as median with interquartile range. The information obtained from the PedsQL™ questionnaire was analysed according to the scoring protocol of the PedsQL™ version 4.0. The Likert scale was reversed scored and linearly transformed to a 0-100 scale as follows: 0=100, 1=75, 2=50, 3=25, 4=0. The composite Health related quality of life (HRQoL) and the HRQoL domains were represented as mean with standard deviation score. The association between independent variables and HRQoL was tested using simple logistic regression, while multivariate analysis was done for variables with significant association with HRQoL. Confidence interval was set at 95% and p value at < 0.05.

**Ethical consideration:** ethical approval was obtained from the Institutional Review Board (IRB) of NIMR prior to commencement of the study. Participation was voluntary, confidentiality was maintained and freedom to withdraw at any point without negative consequences emphasized prior to enrolment.

## Results

**General characteristics of study participants:** a total of 113 children and adolescents participated in the study. Majority were male (53.1%), aged 13-18 years (65.5%), had secondary education (76.1%), and had both parents alive (68.2%). Concerning HIV related characteristics, majority were on first line antiretroviral drugs (70.8%), had been on ART for > 60 months (85.8%), had viral suppression (viral load < 1000 copies/ml) (80.5%) and CD4 lymphocyte count ≥ 500 cells/µL in (78.8%). This is depicted in Table 1 below.

**Table 1:** characteristics of study participants

Characteristics	Frequency (%)
	**N=113**
**Gender**	
Male	60 (53.1%)
Female	53 (46.9%)
M:F ratio	1.1:1
**Age groups [years]**	
8-12	39 (34.5%)
13-18	74 (65.5%)
**Level of education**	
Primary	22 (19.5%)
Secondary	86 (76.1%)
Tertiary	5 (4.4%)
**Orphan status**	
Non orphan	77 (68.1%)
Double orphan	9 (8.0%)
Maternal orphan	14 (12.4%)
Paternal orphan	13 (11.5%)
**ART regimen**	
First line therapy	80 (70.8)
Second line therapy	33 (29.2)
**ART duration**	
60 months	97 (85.8)
≤ 60 months	16 (14.2)
**CD4 lymphocyte count**	
≥500 cells/µL	89 (78.8)
<500 cells/µL	24 (21.2)
**Viral load**	
≥1000 copies/ml	22 (19.5)
<1000 copies/ml	91 (80.5)

**Health-Related Quality of Life [HRQoL] scores of study participants:** concerning the health related quality of life of participants, in the PedsQL^TM^ 4.0, the mean physical health status, psychosocial health status and the total perceived quality of life were 85.0 (±22.4)%, 80.2 (±17.2)%, and 81.6 (±18.4)%, respectively. The perceived total [85.0% ±15.6%], and physical [89.9%±15.9%] quality of life scores of adolescents aged 13-18 years were significantly better than the scores for children aged 8-12 years, while there was no significant difference in the two age groups with regard to their psychosocial scores. This is shown in Table 2.

**Table 2:** health-related quality of life scores of study participants by age group

Characteristic	All Participants	8-12 years	13-18 years	p value*
Physical mean (±SD)	85.0 (±22.4)	75.7 (±29.2)	89.9 (±15.9)	0.0001
Psycho-social mean (±SD)	80.2 (±17.2)	74.3 (±17.6)	80.2 (±17.2)	0.0844
Overall mean (±SD)	81.6 (±18.4)	74.9 (±21.4)	85.0 (±15.6)	0.0049

* p value compares the mean HRQoL of participants aged 8-12years and 13-18years.

**Grades of health-related quality of life scores:** the overall HRQoL scores of the participants were graded as Good (80%-100%), Intermediate (≥ 60% - < 80%) and Poor (<60%). A higher proportion of the age group 13 years to 18 years, and males had good HRQoL grades. Being on second line ART, duration on ART ≥ 60 months, viral suppression and CD4 count ≥ 500 cells/µL were also associated with good HRQoL grades. Parental status had no association with the HRQoL scoring. The grades by participants´ characteristics are shown in Table 3.

**Table 3:** grading of health-related quality of life by participants’ characteristic

Characteristic	Good (n = 78)	Intermediate (n = 19)	Poor (n = 16)	Total (n =113)
**Age groups (years)**				
8-12	22 (56.4%)	9 (23.1%)	8 (20.5%)	39 (100.0%)
13-18	56 (75.7%)	10 (13.5%)	8 (10.8%)	74 (100.0%)
**Gender**				
Male	51 (85.0%)	5 (8.3%)	4 (6.7%)	60 (100.0%)
Female	27 (50.9%)	14 (26.4%)	12 (22.7%)	53 (100.0%)
**Orphan status**				
One/both parents alive	71 (68.3%)	18 (17.3%)	15 (14.4%)	104 (100.0%)
Double orphan	7 (77.8%)	1 (11.1%)	1 (11.1%)	9 (100.0%)
**ART**				
First line	55 (67.9%)	12 (14.8%)	14 (17.3%)	81 (100.0%)
Second line	23 (71.9%)	7 (21.9%)	2 (6.2%)	32 (100.0%)
**Duration of ART**				
≤ 60 months	6 (37.5%)	4 (25.0%)	6 (37.5%)	16 (100.0%)
>6 0months	72 (74.2%)	15 (15.5%)	10 (10.3%)	97 (100.0%)
**Viral load (copies/ml)**				
<1000	66 (72.5%)	14 (15.4%)	11 (12.1%)	91 (100.0%)
≥1000	12 (54.5%)	5 (22.7%)	5 (22.7%)	22 (100.0%)
**CD4 count (cells/µL)**				
≥500	63 (70.8%)	12 (13.5%)	14 (15.7%)	89 (100.0%)
<500	15 (62.5%)	7 (29.2%)	2 (8.3%)	24 (100.0%)

**Association between participant characteristics and HRQoL:** age group 13 years to 18 years (OR = 2.40 [95% CI 1.05-5.49] p = 0.0352), male gender (OR = 5.46 [95% CI 2.24-13.29] p = 0.0009), and duration on ART ≥ 60 months (OR = 4.80 [95% CI 2.24-13.29] p = 0.0032), were all significantly associated with good versus intermediate/poor HRQoL. Although being virally suppressed and having a CD4 count ≥ 500 cells/µL gave higher likelihood of having a good HRQoL, the difference was not statistically significant. Whether the primary caregiver was the parent of the child or not was not associated with HRQoL scoring. This is shown in Table 4.

**Table 4:** association between participant characteristics and HRQoL

Characteristics	Good [Frequency %)]	Intermediate/poor [Frequency (%)]	OR [95% CI]	P value
Age Group (years)	56 (75.7)	18 (24.3)	2.40	
13-18	22 (56.4)	17 (43.7)	[1.05-5.49]	
8-12				0.00009
Gender	51 (85.0)	9 (15.0)	5.46	
Male	27 (50.9)	26 (41.1)	[2.24-13.29]	
Female				0.554
Primary caregiver	71 (68.3)	33 (31.7)	0.61	
≥ One parent	ï¿½ 7 (77.8)	2 (22.2)	[0.12-3.12]	
Non- Parent				0.680
ART regimen	23 (71.9)	9 (28.1)	1.21	
Second line	55 (67.9)	26 (32.1)	[0.49-2.97]	
First line				0.0032
ART duration	72 (74.2)	25 (25.8)	4.80	
>60 months	6 (37.5)	10 (62.5)	[1.58-14.56]	
≤60 months				0.436
CD4 count cells/μL	63 (70.8)	26 (29.2)	1.45	
≥500	15 (62.5)	9 (37.5)	[0.57-3.74]	
<500			2.20	0.102
Viral load (copies/ml)	66 (72.5)	25 (27.5)	[0.85-5.73]	
<1000	12 (54.5)	10 (45.5)		
≥1000				
Multivariate analysis of participant characteristics and good HRQoL	**B**	**OR**	**95% CI**	**p-value**
Characteristic				
Age group	0.192	1.212	1.029-1.428	0.002
13-18 years				
Gender	-1.744	0.175	0.067-0.455	0.001
Male				
Duration on ART	-1.802	0.165	0.044-0.620	0.008

**Multivariate analysis of factors associated with Good HRQoL:** characteristics that had a p value of ≤ 0.1 in relation to good HRQoL scores were further subjected to multivariate analysis, at the end of which age group (13-18 years), male gender, and duration of ART ≥ 60 months retained their significant association with good HRQoL scores. This is outlined in Table 5.

## Discussion

This study set out to evaluate the health related quality of life [HRQoL] of children and adolescents aged 8-18years who are living with HIV in Lagos, Nigeria, using the Paediatric quality of life questionnaire (PedsQL™). With the ultimate aim of treatment of HIV with (HAART) being viral suppression, immune reconstitution and prevention of transmission, the quality of life of infected children and adolescents is often overlooked as the main emphasis is on ensuring adherence to ARVs and clinical wellbeing. However, assessing the HRQoL is important not only for monitoring the impact of the disease, but also in evaluating the outcome of treatment [27].

In the current study, the majority of the participants had at least one parent alive, were on first line ART and had been on treatment for more than 60 months. There was viral suppression and good immunologic status (CD4 counts > 500 cells/µL) in the majority of the participants as well. The overall quality of life of children living with HIV in this study was good, though the perceived physical HRQoL was better than the psychosocial quality of life.

This overall good health related quality of life scores could be due to the good virologic and immunological status of the participants. This good clinical status could be as a result of the comprehensive Paediatric/Adolescent HIV services at our centre, easy access to ARTs at no cost, prompt attention to issues, routine counselling services, social support groups through regular adolescent club meetings and parental support. This finding is in concordance with previous works [14, 28, 29], and in discordance with previous studies before the era of comprehensive HIV care [30-32], thus affirming the benefits of ART accessibility and availability.

The overall mean HRQoL score and the mean physical score in adolescents aged 13 years to 18 years were significantly better than the corresponding scores for children aged 8 years to 12 years. The reasons adduced for this include that adolescents aged to 13 -18 years have generally been on ART for longer, are aware of their HIV status, and are members of the adolescent support group. They are thus more likely to have come to terms with their status and thus have a better outlook on life and a better quality of life. Some younger children have not had their positive HIV status disclosed to them by their caregivers for varying reasons. They are thus excluded from the adolescent support group, as status disclosure is an entry criterion. They are also subjected to daily use of drugs (ARVs) without being informed of the need for the medications, as well as (un) intentional limitation in daily activities by their parents. This may create a perception of being unhealthy in these children compared to the older participants. Though some previous studies found no association between age and HRQoL scores [14, 28, 29], our results are similar to those obtained by other workers who showed that social support services are associated with better HRQoL [33, 34].

In the current work, there was also significant association between male gender and longer duration on ART and good perceived overall health related quality of life. This association with male gender may reflect the perceived superior value, the role and mentality of the male child with respect to prevalent socio-cultural norms in the study setting. It could also be due to the possibility of males finding better ways to combat their problems, and the lower levels of internalizing negative emotions such as anxiety, sadness or other psychosocial issues or physical challenges compared to females [35-37]. However, this will need further evaluation in future studies in children and adolescents. The better HRQoL associated with longer duration on ART is similar to the report of the PREDICT study in Southeast Asia [34]. This may be because of increased likelihood of viral suppression and better clinical outcome, making for a sense of wellbeing and thus better HRQoL.

In contrast, our study showed that being virally suppressed, or having good immunological status (CD4 count > 500 cells/µL) was not significantly associated with good quality of life scores. This is probably because a large majority of participants were virally suppressed and had good immunologic status. Those who were virally unsuppressed or had poorer immune status were thus too few to elicit a significant association. Similarly, having at least one parent as a caregiver showed no significant association with HRQoL scores. This could also be due to the 92% of participants having at least one parent alive. On the other hand, it could be that the double orphans in this study are being cared for in well-to-do households, without the caregiver having to cope with the strains of HIV infection as would be the case in those being cared for by one or both of their parents.

The limitation to the current study includes the cross-sectional nature of the study and our inability to identify causality for those with good or poor health related quality of life. Thus, a longitudinal study/Focus group discussion could be adopted in future studies to identify possible predisposing factor or causes, as well as specific psychosocial issues among the children living with HIV using appropriate tools.

## Conclusion

The study shows good overall HRQoL scores among children and adolescents living with HIV in our centre, Age 13-18 years, being male and being on ART for ≥ 60 months were significantly associated with good HRQoL scores.

### What is known about this topic

- This good clinical status could be as a result of the comprehensive paediatric/adolescent HIV services, where there is easy access to ARTs at no cost, prompt attention to issues, routine counselling services, social support groups through regular adolescent club meetings and parental support;
- Good HRQoL is associated with longer duration on ART.

### What this study adds

- This study adds to knowledge where it was observed that the overall mean HRQoL score and the mean physical score in adolescents aged 13 years to 18 years were significantly better than the corresponding scores for children aged 8 years to 12 years;
- This study adds to knowledge by showing that there was an overall good quality of life in children with HIV also noted was that the physical HRQoL was better than the psychosocial quality of life;
- This study showed that being virally suppressed, or having good immunological status (CD4 count ≥ 500 cells/µL) was not significantly associated with good quality of life scores.
